# Neuroprotective Effects of Açaí (*Euterpe oleracea* Mart.) against Rotenone* In Vitro* Exposure

**DOI:** 10.1155/2016/8940850

**Published:** 2016-10-03

**Authors:** Alencar Kolinski Machado, Ana Cristina Andreazza, Tatiane Morgana da Silva, Aline Augusti Boligon, Vanusa do Nascimento, Gustavo Scola, Angela Duong, Francine Carla Cadoná, Euler Esteves Ribeiro, Ivana Beatrice Mânica da Cruz

**Affiliations:** ^1^Postgraduate Program of Pharmacology, Federal University of Santa Maria, Santa Maria, RS, Brazil; ^2^Biogenomics Laboratory, Department of Morphology, Health Science Center, Federal University of Santa Maria, Santa Maria, RS, Brazil; ^3^Department of Psychiatry, University of Toronto, Toronto, ON, Canada; ^4^Centre for Addiction and Mental Health, Toronto, ON, Canada; ^5^Federal University of Pelotas, Pelotas, RS, Brazil; ^6^Phytochemical Research Laboratory, Department of Industrial Pharmacy, Federal University of Santa Maria, Santa Maria, RS, Brazil; ^7^Third Age Open University, University of Amazonas State, Manaus, AM, Brazil; ^8^Postgraduate Program of Biological Sciences, Toxicological Biochemistry, Federal University of Santa Maria, Santa Maria, RS, Brazil

## Abstract

Neuropsychiatric diseases, such as bipolar disorder (BD) and schizophrenia (SCZ), have a very complex pathophysiology. Several current studies describe an association between psychiatric illness and mitochondrial dysfunction and consequent cellular modifications, including lipid, protein, and DNA damage, caused by cellular oxidative stress.* Euterpe oleracea* (açaí) is a powerful antioxidant fruit. Açaí is an Amazonian palm fruit primarily found in the lowlands of the Amazonian rainforest, particularly in the floodplains of the Amazon River. Given this proposed association, this study analyzed the potential* in vitro* neuropharmacological effect of* Euterpe oleracea* (açaí) extract in the modulation of mitochondrial function and oxidative metabolism. SH-SY5Y cells were treated with rotenone to induce mitochondrial complex I dysfunction and before and after we exposed the cells to açaí extract at 5 *μ*g/mL. Treated and untreated cells were then analyzed by spectrophotometric, fluorescent, immunological, and molecular assays. The results showed that açaí extract can potentially increase protein amount and enzyme activity of mitochondrial complex I, mainly through NDUFS7 and NDUFS8 overexpression. Açaí extract was also able to decrease cell reactive oxygen species levels and lipid peroxidation. We thus suggest açaí as a potential candidate for drug development and a possible alternative BD therapy.

## 1. Introduction

Neuropsychiatric diseases are an important problem in public health around the world [1, 2]. Bipolar disorder (BD) is a chronic mental illness that causes significant impairment in life quality and is an important cause of disability in young people [3, 4]. The prevalence of BD in the world is around 3%, and it can affect populations independently of socioeconomic status or nationality [3]. Patients with BD present recurrent episodes of mania and depression, but the etiology of the disease is still not completely clear. Usually subjects with BD have a genetic component that interacts with the environment to develop the disease [5]. Some evidence suggests the significant role of mitochondria in BD [6–8]. Current research has demonstrated that BD is associated with mitochondrial complex I deficiency and it can decrease ATP production and increase reactive oxygen species (ROS) levels. Consequently, the cells present oxidative stress followed by different cell damage, including lipid peroxidation, protein oxidation, and DNA damage [9, 10]. The brain is one of the tissues most affected by mitochondrial dysfunction due to its high sensitivity to oxidative stress and energy demands for normal neurotransmission [11, 12].

Currently, the pharmacotherapy for BD involves mood stabilizers and second-generation antipsychotics [13–15] whose side effects are proportional to the duration of treatment and dose of medication [4], and these side effects are the main cause of treatment interruption. The prolonged use of different antipsychotic medications has been related to the incidence of metabolic dysfunctions, such as obesity, dyslipidemia, high blood pressure, and increased glycaemia levels [1, 4]. Lithium, the main mood stabilizer used in BD therapy [2], acts directly on cell mitochondria. Hou et al. [16] showed that lithium is able to protect dopaminergic cells against mitochondrial complex I deficiency induced by rotenone exposition; however, lithium can also cause side effects, including memory disorders, renal dysfunction [17–19], and metabolic diseases such as diabetes and hypothyroidism [4]. Therapy for BD is also still limited, because the results obtained from drug development studies are usually unsatisfactory or unsafe, and the action mechanism of some medications is still unclear [2]. Functional foods and natural product studies that could possibly improve mitochondrial function might thus help subjects with BD to recover their quality of life and decrease the extensive burden of this disease.

This is the case of* Euterpe oleracea*, an Amazonian Brazilian fruit popularly known as açaí [20]. Açaí presents several bioactive molecules with different bioactive properties, including antioxidant, anti-inflammatory, and analgesic activities, and it is also able to modulate calcium homeostasis and autophagy on brain cells [21–26]. Açaí's biological effects are related to its chemical matrix, which includes numerous phytochemicals components such as flavonoids [27]. These molecules can neutralize ROS by itself and/or inactivate molecules with prooxidant capacity [28–30]. It is also known that flavonoids have potent anti-inflammatory effects [31, 32]. In an* in vitro* study, Xie et al. [33] demonstrated that velutin, found in açaí fruit, is able to decrease proinflammatory cytokines as tumor necrosis factor- (TNF-) alpha and interleukin- (IL-) 6 in macrophage cells. Another flavonoid found in açaí fruit is apigenin [34]. Apigenin has been identified as a neuroprotective biomolecule against Alzheimer's disease [35], Parkinson disease [36], and ischemic injury [37, 38]. Since açaí is a rich source of flavonoids and other compounds with bioactive power we hypothesize that freeze-dried hydroalcoholic açaí extract might have positive effects against neuropsychiatric diseases as BD.

On this basis, we developed a study of açaí freeze-dried hydroalcoholic extract effects on neuronal-like cells (SH-SY5Y) with mitochondrial complex I deficiency. The main objective of this research was to analyze whether* Euterpe oleracea* extract is able to prevent and/or reverse mitochondrial dysfunction induced by rotenone exposure and also protect against cell imbalance consequences. This study could open new exploratory means of drug development and targets of therapy for BD.

## 2. Materials and Methods

### 2.1. *Euterpe oleracea* Extract and Quantification of Compounds

Fresh açaí fruits were obtained from a harvesting area in Manaus city, Amazonas state. To prevent any change in the fruit quality and properties of the components, they were frozen immediately after the fruit harvest and kept at −20°C. The frozen fruits were transported to the Biogenomics Laboratory at the Federal University of Santa Maria. The fruits were confirmed to be* Euterpe oleracea* by a specialist in plant ecology and botany. A freeze-dried hydroalcoholic extract of açaí was obtained. First of all the açaí fruits were manually macerated to remove the seeds. Then the skin and pulp were placed in ethanol (Neon® commercial-03467; Sao Paulo, SP, Brazil) 70% (70% absolute ethanol : 30% distilled water; v : v) for 21 days at a concentration of 300 mg/mL. After the period of extraction, the material was filtered and the liquid part was lyophilized after ethanol removal. Freeze-dried hydroalcoholic extract powder was then conducted to compounds quantification and characterization.

To determine the main molecules present in açaí hydroalcoholic extract as a chemical matrix we performed the analysis through high performance liquid chromatography (HPLC-DAD) using the Shimadzu Prominence Auto Sampler (SIL-20A) system (Shimadzu, Kyoto, Japan). The freeze-dried hydroalcoholic extract was analyzed following the protocol reported by Klimaczewski et al. [39] at 15 mg/mL. The standards used in this analysis included formic acid, gallic acid, caffeic acid, chlorogenic acid,* p*-coumaric acid, catechin, and epicatechin purchased from Merck (Darmstadt, Germany), chrysin, luteolin, apigenin, orientin, and vitexin, acquired from Sigma Chemical Co. (St. Louis, MO, USA), and cyanidin 3-*0*-glucoside acquired from ChromaDex (Irvine, CA, USA). We calculated the limit of detection (LOD) and the limit of quantification (LOQ) based on the standard deviation of response and the slope using three independent curves of analysis, as described by Abbas et al. [40].

### 2.2. Cell Culture and Treatments

Neuronal-like cells SH-SY5Y were obtained from the American Type Culture Collection (ATCC® CRL-2266*™*; Manassas, VA, USA) and cultured in DMEM/F12 medium (Gibco® Thermo Fisher-11320033; Mississauga, ON, Canada) supplemented with 10% of fetal bovine serum (FBS) (Gibco® Thermo Fisher-12484028; Mississauga, ON, Canada) and 1% penicillin (100 U/mL)/streptomycin (100 mg/mL) (Gibco® Thermo Fisher-15140122; Mississauga, ON, Canada). Cells were cultured until there was an ideal confluence and number of cells to perform all the treatments and experiments at 37°C in 5% CO_2_ and 95% O_2_ in a humidified environment.

The treatments were performed to measure the capacity of açaí freeze-dried hydroalcoholic extract to prevent and/or reverse mitochondrial complex I deficiency and the possible damage caused by this cellular imbalance. To induce mitochondrial complex I deficiency we exposed the cells to rotenone in different concentrations (5, 15, and 30 nM) during 24 h incubation as described by Kim et al. [41]. Before and after rotenone exposure treatment was performed with hydroalcoholic lyophilized açaí extract at optimal cells culture conditions.

### 2.3. Cell Viability Assay

To select the most effective concentration of açaí freeze-dried hydroalcoholic extract in SH-SY5Y cells we measured cell viability response by XTT (tetrazolium salt; Sigma-Aldrich-x4251; St. Louis, MO, USA) assay, following the manufacturer's instructions. The cells were exposed to different concentrations of açaí freeze-dried hydroalcoholic extract (0.001 *μ*g/mL–1000 *μ*g/mL) under different periods of incubation (24, 48, and 72 hours). The most effective freeze-dried extract concentration and time of incubation treatment were selected calculating the EC50 to perform all the other assays of this research, adding açaí freeze-dried hydroalcoholic extract before or after rotenone exposure.

### 2.4. Scanning Confocal Microscopy Analysis of Cell Morphology

SH-SY5Y cells treated with rotenone and/or açaí freeze-dried hydroalcoholic extract were placed under sterile glass cover lids (VWR Collection-2441; Radnor, PA, USA) in 6-well cell culture plates with 7.5 × 10^4^ cells/well overnight to evaluate cell morphology. Cells were treated with rotenone and/or açaí freeze-dried hydroalcoholic extract according to the experimental designs performed for cell viability analysis. After all treatments the cells were fixed with ethanol : acetic acid (3 : 1, v : v). Images were acquired using a fluorescence microscope (Nikon Eclipse Ti-U; Mississauga, ON, Canada) and a high sensitivity QImaging camera, model Retiga*™* 1300 (QImaging Scientific cameras; Surrey, BC, Canada) at 20x magnification.

### 2.5. Analysis of Human Mitochondrial Oxidative Phosphorylation (OXPHOS)

The oxidative phosphorylation of neuronal-like cells previously treated with rotenone and/or açaí freeze-dried hydroalcoholic extract for both research elements (prevent and reverse) was measured through a human oxidative phosphorylation magnetic bead panel (Millipore, H0XPSMAG-16K; Toronto, ON, Canada) following the manufacturer's instructions. In this assay we analyzed all the mitochondrial complexes (I, II, III, IV, and V) simultaneously in an individual well reaction per treatment in triplicate. Results were obtained using the high precision machine Megapix® (Luminex Corporation xMAP Technology; Toronto, ON, Canada).

### 2.6. Mitochondrial Complex I Enzyme Activity

We measured mitochondrial NADH: ubiquinone oxidoreductase complex (mitochondrial complex I) activity using a mitochondrial complex I enzyme activity kit (Abcam-ab-109721; Cambridge, UK) following the manufacturer's instructions. This is a colorimetric assay based on enzyme immunocapture through the oxidation of NADH to NAD+.

### 2.7. Protein Expression of Mitochondrial Complex I Subunits

To analyze the effect of açaí on the amount of protein in the mitochondrial complex I Q module subunits NDUFS7 and NDUFS8 and also in N module subunits NDUFV1 and NDUFV2, four very important subunits associated with complex I activity, we performed western blot analysis according to a protocol reported by Andreazza et al. [42]. The cell lysate of each treatment was loaded on to 12% polyacrylamide (Sigma-Aldrich-M7279; St. Louis, MO, USA) gels and transferred to polyvinylidene difluoride (PVDF) membranes (GE Healthcare-10600023; Little Chalfont, UK). The membranes were stained with Memcode reversible stain (Thermo Fisher-23227; Mississauga, ON, Canada) according to the manufacturer's protocol. After a blocking process (1 h, 5% BSA) the membranes were incubated with primary anti-NDUFS7, anti-NDUFS8, anti-NDUFV1, or anti-NDUFV2 (Santa Cruz Biotechnology-98644; Dallas, USA) (Abcam-ab96123; Cambridge, UK) rabbit polyclonal antibodies (1 : 1000). After 90 minutes of incubation we added the secondary anti-rabbit (1 : 1000). Membranes were incubated with ECL western blotting substrate (Thermo Fisher-32106; Mississauga, ON, Canada) and analyzed by acquiring images using Versa Doc equipment 5000 MP (Bio-Rad Laboratories Ltd., Mississauga, ON, Canada) and Quantity One Analysis software version 4.6.9 (Bio-Rad Laboratories Ltd., Mississauga, ON, Canada). Sample intensity was normalized by beta-actin protein levels.

### 2.8. Gene Expression of Mitochondrial Complex I Subunits Genes

To complement mitochondrial complex I analysis, we measured NDUFS7, NDUFS8, NDUFV1, and NDUFV2 gene expression using qRT-PCR. Initially the RNA was extracted from treated cells using Trizol® reagent (Thermo Fischer-15586026; Mississauga, ON, Canada). After RNA quantification through a NanoDrop*™* 1000 Spectrophotometer System® (Thermo Scientific, Wilmington, DE, USA), RNA was converted to complementary DNA (cDNA) using a QuantiTect® Reverse Transcription Kit (Qiagen-205311; Toronto, ON, Canada). Real-time PCR was performed using NDUFS7, NDUFS8, NDUFV1, or NDUFV2 QuantiTect® Primers (Qiagen-cat. QT0045850; Toronto, ON, Canada) and QuantiFast® SYBR® Green PCR Kit (Qiagen-cat. 204054; Toronto, ON, Canada) on CFX 96*™* Real-Time PCR Detection System (Bio-Rad, Hercules, CA, USA). Beta-actin was used as the housekeeping gene to normalize the gene expression of all samples.

### 2.9. Total Levels of ROS Measurement

Total levels of ROS were determined in treated SH-SY5Y cells using 2,7 dichlorodihydrofluorescein diacetate (DCFH-DA) assay, as described by Costa et al. [43]. DCFH-DA (Sigma-Aldrich-D6883; St. Louis, MO, USA) is a nonfluorescent compound that is deacetylated by mitochondrial esterase enzymes to DCFH which reacts with ROS molecules and becomes DCF, a fluorescent compound. Fluorescence was measured at an excitation wavelength of 488 nm and an emission wavelength of 525 nm.

### 2.10. Lipid Peroxidation Analysis

Lipid peroxidation was analyzed using thiobarbituric acid reactive substances (TBARS) kit assay (Cayman Chemical-700870; Ann Arbor, MI, USA), following the instructions described by the manufacturer. The malondialdehyde (MDA)/thiobarbituric acid reaction was determined measuring the absorbance at 535 nm.

### 2.11. Statistical Analysis

The data was first transformed to percentages against a negative control group. Results from dose-response curves were statistically analyzed using one-way ANOVA, and other results were analyzed using two-way ANOVA analysis of variance. Both were followed by a Tukey* post hoc* test, using Graphpad prism software, version 5.0 (Graphpad Prism software, 2015; San Diego, CA, USA). Results of *p* < 0.05 were considered significant.

## 3. Results

**Figure 1:**
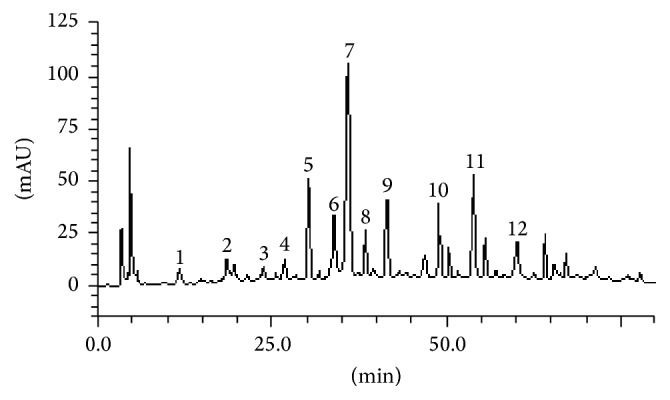
Representative high performance liquid chromatography profile of* Euterpe oleracea* freeze-dried hydroalcoholic extract. Gallic acid (peak 1), catechin (peak 2), chlorogenic acid (peak 3), caffeic acid (peak 4),* p*-coumaric acid (peak 5), epicatechin (peak 6), orientin (peak 7), vitexin (peak 8), cyanidin-3-*0*-glucoside (peak 9), luteolin (peak 10), apigenin (peak 11), and chrysin (peak 12).

Twelve molecules were quantified in the açaí freeze-dried hydroalcoholic extract: gallic acid (retention time, Rt = 10.27 min; peak 1), catechin (Rt = 17.83 min; peak 2), chlorogenic acid (Rt = 23.91 min; peak 3), caffeic acid (Rt = 26.11 min; peak 4),* p*-coumaric acid (Rt = 29.97 min; peak 5), epicatechin (Rt = 34.08 min; peak 6), orientin (Rt = 35.14 min; peak 7), vitexin (Rt = 37.41 min; peak 8), cyanidin-3-*0*-glucoside (Rt = 41.65 min; peak 9), luteolin (Rt = 48.25 min; peak 10), apigenin (Rt = 54.13 min; peak 11), and chrysin (Rt = 60.27 min; peak 12) (Figure 1 and Table 1). The three most concentrated molecules found in the extract were orientin (8.05 ± 0.03),* p*-coumaric acid (3.52 ± 0.01), and apigenin (3.49 ± 0.01).

**Figure 2:**
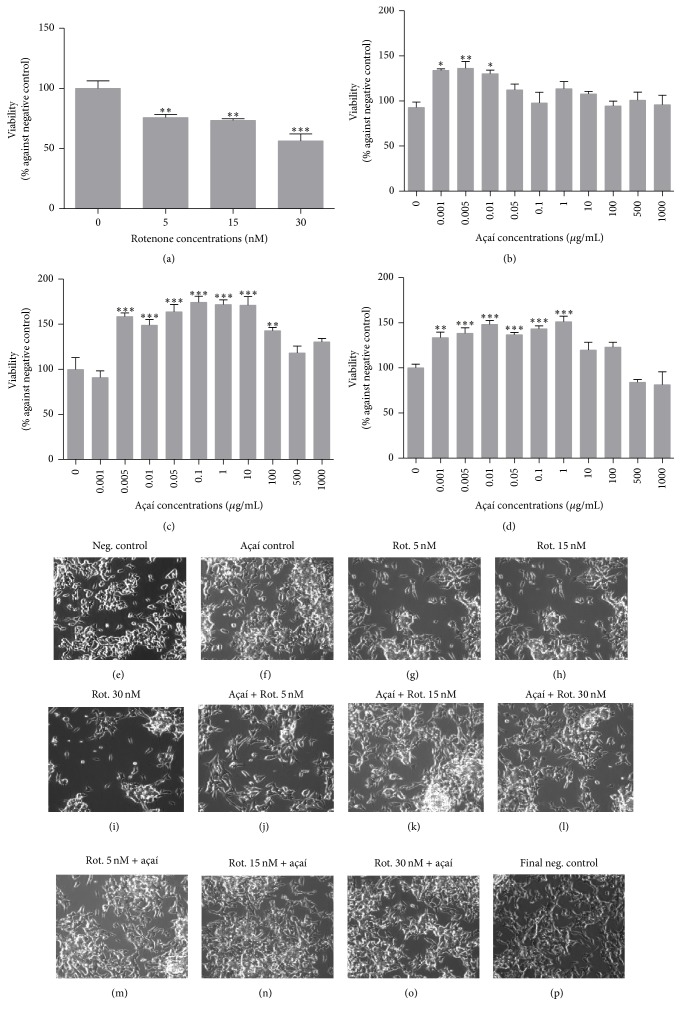
Cell viability measurements. (a) SH-SY5Y cells exposure to 5, 15, and 30 nM of rotenone during 24 h showing significant cell mortality for all concentrations; (b, c, and d) SH-SY5Y cells exposure to different concentrations of açaí freeze-dried hydroalcoholic extract during 24, 48, and 72 h showing a hormetic cell response; (e–p) microscopic scanning of SH-SY5Y cells exposure to rotenone and/or açaí freeze-dried hydroalcoholic extract (5 *μ*g/mL) showing the cytotoxic effect of rotenone and the protective action of açaí freeze-dried hydroalcoholic extract. ^*∗*^ *p* < 0.05; ^*∗∗*^ *p* < 0.01; ^*∗∗∗*^ *p* < 0.001.

The cell viability response of neuronal-like SH-SY5Y cells to different concentrations of açaí freeze-dried hydroalcoholic extract was analyzed (Figure 2). Initially, the cell response to rotenone treatments at 5, 15, and 30 nM was also measured to confirm the toxicity of these concentrations. Cell morphology and rate of proliferation were also analyzed under rotenone and/or açaí freeze-dried hydroalcoholic extract treatments. As expected, rotenone treatments decreased cell viability in a dose-dependent way with a rate of mortality about 24% at 5 nM, 26% at 15 nM, and 43% at 30 nM of rotenone (Figure 2(a)). On the other hand, there was a hormetic response of neuronal-like cells under açaí extract concentrations mainly after 48 and 72 h of treatment (Figures 2(c) and 2(d)) increasing the cell viability rate from 0,005 *μ*g/mL until 100 *μ*g/mL of açaí freeze-dried extract. The açaí extract significantly increased cell viability, mainly after 48 h of incubation, compared to the negative control. We determined the EC50 of açaí freeze-dried hydroalcoholic extract for this dose-response curve, and the value obtained was 5 *μ*g/mL. All other experiments were performed using this specific concentration and period of incubation for the açaí freeze-dried extract.

Scanning confocal microscopy analysis showed that cell morphology was preserved even at rotenone treatments; however cell proliferation decreased considerably at these conditions of exposition (Figures 2(g), 2(h), and 2(i)). The concomitant açaí freeze-dried hydroalcoholic extract exposition at 5 *μ*g/mL (Figure 2(f)) retained cell morphology and recovered the proliferation of cells exposed to different concentrations of rotenone (Figures 2(j)–2(o)) similarly to the final negative control (Figure 2(p)). In both experimental designs (prevent and reverse) açaí extract was able to stimulate cell proliferation compared to the negative control, neutralizing the rotenone effect.

**Figure 3:**
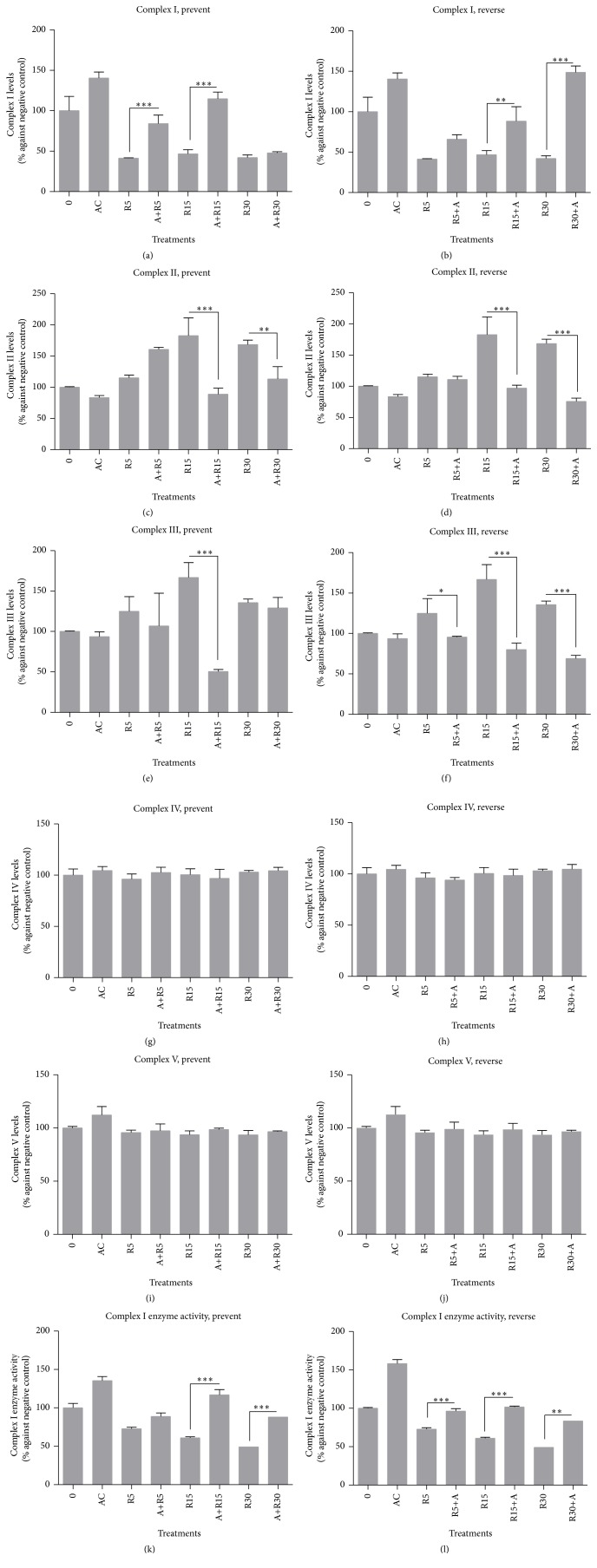
Cellular oxidative phosphorylation pathway measurements. (a–j) Rotenone exposition decreased mitochondrial complex I and increased mitochondrial complexes II and III, and açaí freeze-dried hydroalcoholic extract normalized the protein expressions. (k and l) Rotenone decreased mitochondrial complex I enzyme activity and açaí freeze-dried hydroalcoholic extract was able to increase enzyme activity in both designs of experiment. ^*∗*^ *p* < 0.05; ^*∗∗*^ *p* < 0.01; ^*∗∗∗*^ *p* < 0.001.

The capacity of açaí to prevent and/or reverse mitochondrial function was evaluated from these results. The OXPHOS analysis showed a decreased protein expression for mitochondrial complex I and açaí freeze-dried hydroalcoholic extract presented an increased potential. Açaí supplementation at 5 *μ*g/mL also improved the mitochondrial complex I proteins of neuronal-like cells in both experimental designs, mainly preventing the rotenone effects of 5 and 15 nM (Figure 3(a)) and reversing those of 15 and 30 nM (Figure 3(b)). On the other hand, we observed the opposite response for mitochondrial complexes II and III under rotenone treatments and in this case, açaí freeze-dried hydroalcoholic extract proved to cause protein expression in similar conditions to the negative control at 15 and 30 nM of rotenone for both experimental designs (Figures 3(c), 3(d), 3(e), and 3(f)). No significant results were observed for mitochondrial complexes IV and V (Figures 3(g), 3(h), 3(i), and 3(j)).

Mitochondrial complex I enzyme activity presented a decreased dose-dependent activity under rotenone treatments and an increased enzyme action under açaí exposure. Açaí proved to be able to improve the enzyme activity of this complex under preventative and reverse conditions, especially before and after rotenone 15 nM (Figures 3(k) and 3(l)).

**Figure 4:**
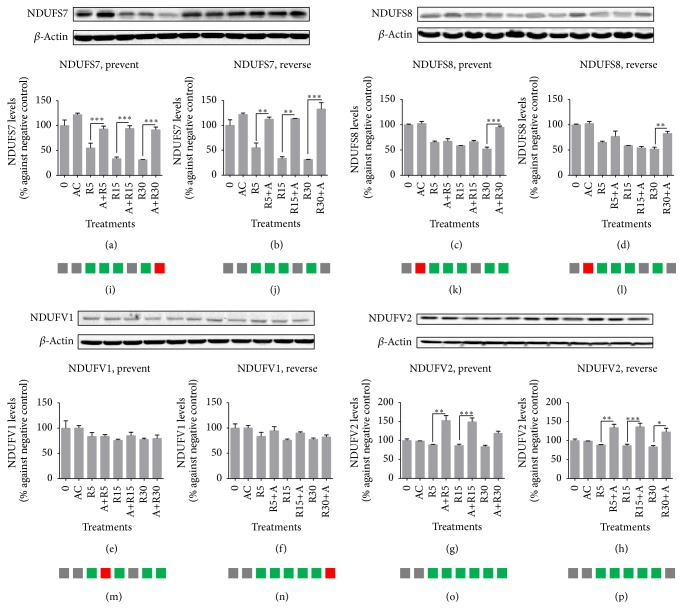
Mitochondrial complex I subunits analysis by western blot and qRT-PCR assays. The order of treatment for all the blot images: 0, Rot. 5, Rot. 15, Rot. 30, A+R5, A+R15, A+R30, R5+A, R15+A, and R30+A; (a and b) NDUFS7 protein expression analysis; (c and d) NDUFS8 protein expression analysis; (e and f) NDUFV1 protein expression analysis; (g and h) NDUFV2 protein expression analysis. Gene expression analysis follows the same graph order where gray means normal gene expression, green means downregulation gene expression, and red means upregulation gene expression. (i and j) NDUFS7 gene expression analysis; (k and l) NDUFS8 gene expression analysis; (m and n) NDUFV1 gene expression analysis; (o and p) NDUFV2 gene expression analysis. ^*∗*^ *p* < 0.05; ^*∗∗*^ *p* < 0.01; ^*∗∗∗*^ *p* < 0.001.

Açaí freeze-dried hydroalcoholic extract modulated NDUFS7, NDUFS8, and NDUFV2 protein expression differently, as shown in Figure 4. Rotenone significantly decreased all the subunits tested in this study at all concentrations unless NDUFV1. On the other hand, açaí extract improved protein expression tested in at least one design of the experiment and in one concentration of rotenone for NDUFS7, NDUFS8, and NDUFV2. qRT-PCR for those mitochondrial complex I subunits showed a similar profile for gene and protein expression for NDUFS7 and NDUFS8 (Figures 4(i), 4(j), 4(k), and 4(l)); however, açaí extract modified gene expression differently to protein expression finds, with significant improvements in gene expression for NDUFV1 (Figures 4(m) and 4(n)), which was not observed with western blot analysis.

**Figure 5:**
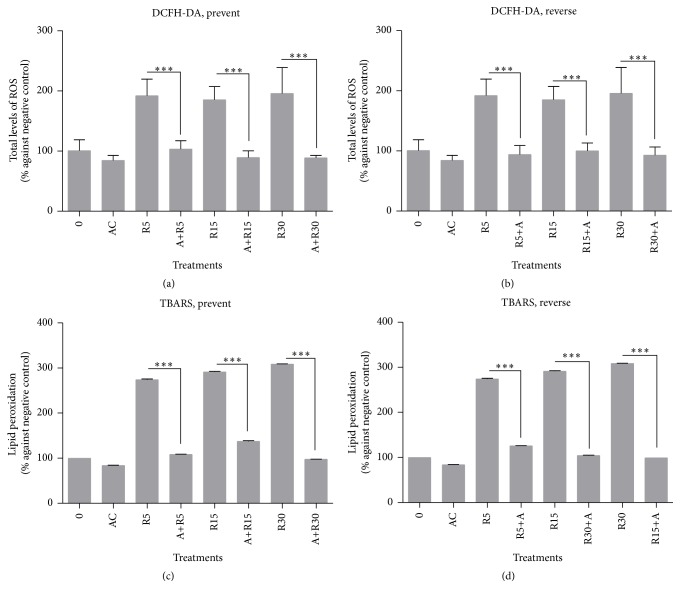
Cell oxidative metabolism biomarker measurements. (a and b) Total levels of ROs measured by DCFH-DA assay; (c and d) lipid peroxidation measured by TBARS assay. While rotenone increased ROs levels in a dose-dependent way and also lipid peroxidation, açaí freeze-dried hydroalcoholic extract was able to decrease both biomarkers compared to the negative control. ^*∗*^ *p* < 0.05; ^*∗∗*^ *p* < 0.01; ^*∗∗∗*^ *p* < 0.001.

While rotenone treatments increased ROS total levels at SH-SY5Y cells in a dose-dependent way, açaí freeze-dried hydroalcoholic extract at 5 *μ*g/mL considerably reduced ROS production for all rotenone concentrations in both experimental designs (Figures 5(a) and 5(b)), showing important antioxidant activity. As a consequence of oxidative stress, rotenone also increased lipid peroxidation tested by TBARS assay; however, as previously expected açaí freeze-dried hydroalcoholic extract was also able to decrease lipid peroxidation rates in both experimental designs (Figures 5(c) and 4(d)), normalizing this imbalance compared to rotenone concentrations and negative control.

## 4. Discussion

**Figure 6:**
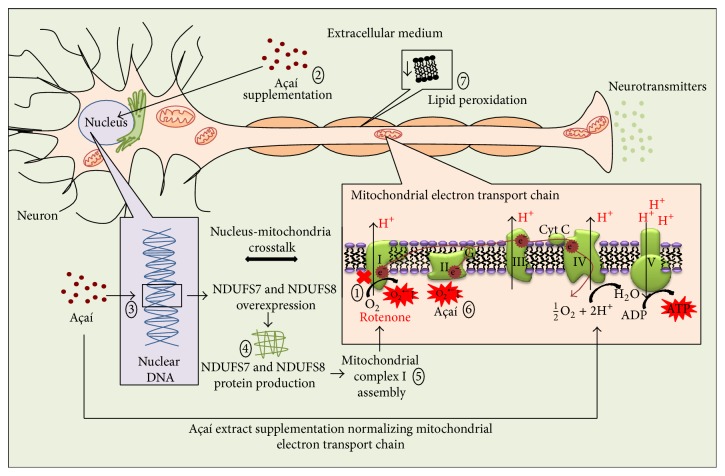
Protective effects of açaí supplementation in neuron-like cells SH-SY5Y exposed to rotenone. ① Rotenone causing mitochondrial complex I dysfunction, increasing superoxide production, and decreasing ATP synthesis; ② açaí freeze-dried hydroalcoholic extract supplementation acting at cell nucleus; ③ açaí freeze-dried hydroalcoholic extract increasing NDUFS7 and NDUFS8 gene expression in particular; ④ NDUFS7 and NDUFS8 protein production in response to the gene overexpression; ⑤ mitochondrial complex I assembly by new protein subunits; ⑥ renormalization of the mitochondrial electron transport chain, decreasing the oxidative stress, and normalizing ATP synthesis. ⑦ Decreased lipid peroxidation in consequence of the oxidative metabolism balance recovery.

The present* in vitro* study described the important protective effects of açaí in neuronal-like SH-SY5Y cells exposed to rotenone that caused mitochondrial dopaminergic dysfunction. The açaí protection involved a reversion of mitochondrial complex I dysfunction and oxidative stress caused by rotenone (Figure 6).

It is currently known that BD is associated with mitochondrial dysfunction, especially at complex I, and this abnormality has several cell oxidative consequences [8, 9, 44]. Since the pharmacotherapy used for BD is neither fully effective nor safe, it is a necessity to search for new alternative therapies. The results described here indicate that the chemical matrix found in açaí fruit could be a potential candidate for improving the function of mitochondrial complex I and subsequently improving neuropsychiatric BD symptoms [21–23].

Açaí freeze-dried hydroalcoholic extract showed the presence of different important compounds with known biological effects, mainly orientin,* p*-coumaric acid, and apigenin. Orientin is known to be an important phenolic compound found in different fruits. This molecule has significant antioxidant, anti-inflammatory, and neuroprotective effects [45–48]. In a study performed by An et al. [49], orientin demonstrated significant antioxidant activity and neural ultrastructure improvement in aged mice. The* p*-coumaric acid is another phenolic compound with considerable antioxidant capacity [50]; however, apigenin also demonstrates antioxidant activity as an important neuroprotective competence due to its ability to cross the blood-brain barrier [51]. Previous studies have shown that apigenin has no toxic effects, even at high doses [52].

It is still unknown whether açaí could cross the blood-brain barrier by itself; however, there are some studies describing its neuroprotective effects. In work developed by Poulose et al. [24], for example, açaí was able to attenuate calcium dysregulation in rodent brain cells and to modulate cell autophagy. Moreover, it is already known that different bioactive compounds, especially flavonoids, can cross the blood-brain barrier [53–55]. In this sense, considering that the chemical matrix of açaí is composed of different flavonoids, perhaps these molecules are able to reach the brain. It is necessary to perform* in vivo* studies to test this hypothesis.

As expected, rotenone decreased SH-SY5Y cell viability in a dose-responsive way as a cytotoxic effect of this chemical, corroborating the finds of Kim et al. [41]. Açaí freeze-dried hydroalcoholic extract increased cell viability measured by XTT assay in a hormetic response. The best rate of cell proliferation was observed at 48 h of incubation and the EC50 for this period of exposition was 5 *μ*g/mL. In a study performed by Wong et al. [56] using rat pheochromocytoma cells, açaí freeze-dried hydroalcoholic extract showed significant neuronal protection against beta-amyloid peptide exposure, increasing cell viability, especially at 5 and 50 *μ*g/mL of açaí freeze-dried hydroalcoholic extract, also supporting our finds.

Rotenone is known to be a reagent able to directly inhibit mitochondrial complex I [57]. In a complex I dysfunction the mitochondria electron transport chain will try to maintain energy production by using complexes II and III which explains their protein overexpression found in this study by OXPHOS assay. Proving an affinity between açaí freeze-dried hydroalcoholic extract and mitochondrial complex I we also observed the significant effect of our extract against rotenone-induced dysfunction, showing not only a protein expression improvement but also a mitochondrial complex I enzymatic activity enhancement. Açaí 5 *μ*g/mL exposition was able to recover mitochondrial complex II and III protein expression, recovering the mitochondrial electron transport chain function. Our results thus suggest a pharmacological effect of açaí freeze-dried hydroalcoholic extract on mitochondrial complex I.

The effects of açaí on the prevention and reversion of mitochondrial complex I dysfunction have been not associated with other plants or pharmacological drugs. For example, in a study developed with rat E18 cortical neurons, Scola et al. [58] showed that lithium at 0.75 mM was able to increase complex I activity compared to rotenone treatments; however lithium treatment was unable to recover 100% of the activity of this complex compared to the negative control. Our findings demonstrate that açaí can increase mitochondrial complex I activity under rotenone-induced mitochondrial dysfunction and also recover 100% or more of the complex I activity, compared to the negative control, renormalizing mitochondrial function.

We measured NDUFS7, NDUFS8, NDUFV1, and NDUFV2 protein and gene expression at SH-SY5Y cell after rotenone and/or açaí treatments. The results showed a significant decrease in their expressions with rotenone exposure; however, açaí increased these expressions in some experimental designs and rotenone concentrations. The main complex I subunit in which açaí presented a pharmacological effect of prevention and reversion of rotenone effects was NDUFS7, measured by both western blot and qRT-PCR. It is already known that rotenone is able to deactivate mitochondrial complex I electron transfer to ubiquinone, meaning mitochondrial complex I Q module (formed by NDUFS7, NDUFS8, NDUFS2, and NDUFS3 subunits) inactivation. Rotenone can also impair complex I transcriptions through direct effects and by induction of the oxidative stress that causes DNA damage and affects the translocation of important transcription factors [58, 59]. Our results showed that açaí has the capacity to improve the gene expression of different nuclear genes. It is also already known that açaí can modulate the gene expression of antioxidant enzymes, including SOD, CAT, and GPx [58]; however, the way in which açaí is able to modulate gene expression positively has not yet been determined. One potential hypothesis is that açaí could increase the transcription factor involved in nuclear gene expression located at promoter regions, such as NRF2, by flavone interaction and activation [60]. The results described here suggest that açaí freeze-dried hydroalcoholic extract acts mainly at the complex I Q module under mitochondrial dysfunction, especially at the NDUFS7 subunit, and this effect is associated with its gene overexpression.

Although rotenone treatments were able to increase oxidative stress by elevation of ROS production and lipid peroxidation, açaí freeze-dried hydroalcoholic extract was able to decrease the levels of both biomarkers for all rotenone concentrations. These results were found in both experimental designs. We postulate that açaí extract can act as an antioxidant agent under mitochondrial complex I deficiency, recovering mitochondrial function and neutralizing cell damage caused by oxidative imbalance.

It is also important to emphasize that açaí did not demonstrate any* in vitro* neurotoxic effect in SH-SY5Y cells.* Annona muricata* fruit, for example, has an important neurotoxic effect because of its chemical matrix which includes polyketides specific to Annonaceae. These metabolites are the most potent mitochondrial complex I inhibitor found in nature, inducing neuronal death [61]. Otherwise, in this study, açaí modulated positively different cellular aspects preserving neuronal viability.

All the results noted in this study are based on* in vitro* experiments. Due to methodological limitations associated with* in vitro* studies, complementary studies using* in vivo* experimental models need to be performed to confirm the evidence described here.

## 5. Conclusions

There are many studies that describe the different activities of plant extracts; however, the effects of fruits and other functional foods on mitochondrial function have not yet been well evaluated. Our results showed that açaí hydroalcoholic extract has an important affinity to mitochondrial complex I. Açaí is able to recover the mitochondrial electron transport chain function of neuronal-like cells under mitochondrial complex I dysfunction mainly through overexpression of important nuclear mitochondrial complex I subunits genes and improvement in their proteomic expression. Our findings suggest that açaí freeze-dried hydroalcoholic extract has a significant pharmacological capacity. Açaí could be a new alternative for drug development research for neuropsychiatric diseases, as BD, mainly as a result of the isolated chemical matrix of this fruit. It is important to note that all findings described here are limited to* in vitro* studies and they may be confirmed by an* in vivo* experimental model in a future study.

## Figures and Tables

**Table 1:** Components of hydroalcoholic *Euterpe oleracea* freeze-dried hydroalcoholic extract.

Compounds	*Euterpe oleracea*		LOD	LOQ
	Fruits (mg/g)	%	*μ*g/mL	*μ*g/mL
Gallic acid	0.73 ± 0.01^a^	0.07	0.011	0.036
Catechin	0.75 ± 0.03^a^	0.07	0.028	0.093
Chlorogenic acid	0.41 ± 0.01^b^	0.04	0.023	0.075
Caffeic acid	0.76 ± 0.01^a^	0.07	0.008	0.026
*p*-Coumaric acid	3.52 ± 0.01^c^	0.35	0.031	0.102
Epicatechin	2.37 ± 0.02^d^	0.23	0.024	0.079
Orientin	8.05 ± 0.03^e^	0.80	0.016	0.053
Vitexin	2.19 ± 0.01^f^	0.21	0.015	0.048
Cyanidin-3-*0*-glucoside	2.62 ± 0.01^g^	0.26	0.009	0.029
Luteolin	2.57 ± 0.02^g^	0.25	0.021	0.070
Apigenin	3.49 ± 0.01^c^	0.34	0.027	0.089
Chrysin	1.83 ± 0.01^h^	0.18	0.013	0.043

Açaí freeze-dried hydroalcoholic extract compound determination. Results are expressed as mean ± standard deviations (SD) of three determinations. Averages followed by different letters differ by Tukey test at *p* < 0.05.

## References

[1] Epstein I., Szpindel I., Katzman M. A. (2014). Pharmacological approaches to manage persistent symptoms of major depressive disorder: rationale and therapeutic strategies. *Psychiatry Research*.

[2] Millan M. J., Goodwin G. M., Meyer-Lindenberg A., Ove Ogren S. (2015). Learning from the past and looking to the future: emerging perspectives for improving the treatment of psychiatric disorders. *European Neuropsychopharmacology*.

[3] Alonso J., Petukhova M., Vilagut G. (2011). Days out of role due to common physical and mental conditions: results from the WHO World Mental Health surveys. *Molecular Psychiatry*.

[4] Grande I., Berk M., Birmaher B., Vieta E. (2016). Bipolar disorder. *The Lancet*.

[5] Craddock N., Sklar P. (2013). Genetics of bipolar disorder. *The Lancet*.

[6] Clay H. B., Sillivan S., Konradi C. (2011). Mitochondrial dysfunction and pathology in bipolar disorder and schizophrenia. *International Journal of Developmental Neuroscience*.

[7] Manji H., Kato T., Di Prospero N. A. (2012). Impaired mitochondrial function in psychiatric disorders. *Nature Reviews Neuroscience*.

[8] Scola G., Kim H. K., Young L. T., Andreazza A. C. (2013). A fresh look at complex I in microarray data: clues to understanding disease-specific mitochondrial alterations in bipolar disorder. *Biological Psychiatry*.

[9] Andreazza A. C., Shoo L., Wang J.-F., Trevor Young L. (2010). Mitochondrial complex I activity and oxidative damage to mitochondrial proteins in the prefrontal cortex of patients with bipolar disorder. *Archives of General Psychiatry*.

[10] Berk M., Kapczinski F., Andreazza A. C. (2011). Pathways underlying neuroprogression in bipolar disorder: focus on inflammation, oxidative stress and neurotrophic factors. *Neuroscience & Biobehavioral Reviews*.

[11] Akarsu S., Torun D., Bolu A. (2014). Mitochondrial complex I and III gene mRNA levels in schizophrenia, and their relationship with clinical features. *Journal of Molecular Psychiatry*.

[12] Beal M. F. (2002). Oxidatively modified proteins in aging and disease. *Free Radical Biology and Medicine*.

[13] Curran G., Ravindran A. (2014). Lithium for bipolar disorder: a review of the recent literature. *Expert Review of Neurotherapeutics*.

[14] Grunze H., Vieta E., Goodwin G. M. (2010). The World Federation of Societies of Biological Psychiatry (WFSBP) guidelines for the biological treatment of bipolar disorders: update 2010 on the treatment of acute bipolar depression. *The World Journal of Biological Psychiatry*.

[15] Goodwin G. M. (2009). Evidence-based guidelines for treating bipolar disorder: revised second edition-recommendations from the British association for psychopharmacology. *Journal of Psychopharmacology*.

[16] Hou L., Xiong N., Liu L. (2015). Lithium protects dopaminergic cells from rotenone toxicity via autophagy enhancement. *BMC Neuroscience*.

[17] Kleiner J., Altshuler L., Hendrick V., Hershman J. M. (1999). Lithium-induced subclinical hypothyroidism: review of the literature and guidelines for treatment. *The Journal of Clinical Psychiatry*.

[18] Markowitz G. S., Radhakrishnan J., Kambham N., Valeri A. M., Hines W. H., D'Agati V. D. (2000). Lithium nephrotoxicity: a progressive combined glomerular and tubulointerstitial nephropathy. *Journal of the American Society of Nephrology*.

[19] Young A. H., Newham J. I. (2006). Lithium in maintenance therapy for bipolar disorder. *Journal of Psychopharmacology*.

[20] Kang J., Thakali K. M., Xie C. (2012). Bioactivities of açaí (*Euterpe precatoria* Mart.) fruit pulp, superior antioxidant and anti-inflammatory properties to *Euterpe oleracea* Mart.. *Food Chemistry*.

[21] Jensen G. S., Wu X., Patterson K. M. (2008). In vitro and in vivo antioxidant and anti-inflammatory capacities of an antioxidant-rich fruit and berry juice blend. Results of a pilot and randomized, double-blinded, placebo-controlled, crossover study. *Journal of Agricultural and Food Chemistry*.

[22] Poulose S. M., Bielinski D. F., Carey A., Schauss A. G., Shukitt-Hale B. (2016). Modulation of oxidative stress, inflammation, autophagy and expression of Nrf2 in hippocampus and frontal cortex of rats fed with açaí-enriched diets. *Nutritional Neuroscience*.

[23] Sun X., Seeberger J., Alberico T. (2010). Açai palm fruit (*Euterpe oleracea* Mart.) pulp improves survival of flies on a high fat diet. *Experimental Gerontology*.

[24] Poulose S. M., Fisher D. R., Bielinski D. F. (2014). Restoration of stressor-induced calcium dysregulation and autophagy inhibition by polyphenol-rich açaí (*Euterpe* spp.) fruit pulp extracts in rodent brain cells invitro. *Nutrition*.

[25] Carey A. N., Miller M. G., Fisher D. R. (2015). Dietary supplementation with the polyphenol-rich açaí pulps (*Euterpe oleracea* Mart. and *Euterpe precatoria* Mart.) improves cognition in aged rats and attenuates inflammatory signaling in BV-2 microglial cells. *Nutritional Neuroscience*.

[26] Poulose S. M., Fisher D. R., Larson J. (2012). Anthocyanin-rich açai (*Euterpe oleracea* Mart.) fruit pulp fractions attenuate inflammatory stress signaling in mouse brain BV-2 microglial cells. *Journal of Agricultural and Food Chemistry*.

[27] Odendaal Y. A., Schauss A. G., Watson R. R., Reedy V. R., Zibadi S. (2014). Potent antioxidant and anti-inflammatory flavonoids in the nutrient-rich Amazonian palm fruit, açaí (Euterpe spp.). *Polyphenols in Human Health and Disease*.

[28] Solanki I., Parihar P., Parihar M. S. (2016). Neurodegenerative diseases: from available treatments to prospective herbal therapy. *Neurochemistry International*.

[29] Schauss A. G., Wu X., Prior R. L. (2006). Antioxidant capacity and other bioactivities of the freeze-dried Amazonian palm berry, *Euterpe oleraceae* Mart. (Acai). *Journal of Agricultural and Food Chemistry*.

[30] Schauss A. G., Wu X., Prior R. L. (2006). Phytochemical and nutrient composition of the freeze-dried amazonian palm berry, *Euterpe oleraceae* Mart. (Acai). *Journal of Agricultural and Food Chemistry*.

[31] García-Lafuente A., Guillamón E., Villares A., Rostagno M. A., Martínez J. A. (2009). Flavonoids as anti-inflammatory agents: implications in cancer and cardiovascular disease. *Inflammation Research*.

[32] González-Gallego J., Sánchez-Campos S., Tuñón M. J. (2007). Anti-inflammatory properties of dietary flavonoids. *Nutricion Hospitalaria*.

[33] Xie C., Kang J., Li Z. (2012). The açaí flavonoid velutin is a potent anti-inflammatory agent: blockade of LPS-mediated TNF-*α* and IL-6 production through inhibiting NF-*κ*B activation and MAPK pathway. *Journal of Nutritional Biochemistry*.

[34] Nicholas C., Batra S., Vargo M. A. (2007). Apigenin blocks lipopolysaccharide-induced lethality in vivo and proinflammatory cytokines expression by inactivating NF-*κ*B through the suppression of p65 phosphorylation. *The Journal of Immunology*.

[35] Zhao L., Wang J.-L., Liu R., Li X.-X., Li J.-F., Zhang L. (2013). Neuroprotective, anti-amyloidogenic and neurotrophic effects of apigenin in an Alzheimer's disease mouse model. *Molecules*.

[36] Patil S. P., Jain P. D., Sancheti J. S., Ghumatkar P. J., Tambe R., Sathaye S. (2014). Neuroprotective and neurotrophic effects of Apigenin and Luteolin in MPTP induced parkinsonism in mice. *Neuropharmacology*.

[37] Cai M., Ma Y., Zhang W. (2016). Apigenin-7-0-*β*-d-(-6^″^-p-coumaroyl)-glucopyranoside treatment elicits neuroprotective effect against experimental ischemic stroke. *International Journal of Biological Sciences*.

[38] Yamagata K., Kitazawa T., Shinoda M., Tagawa C., Chino M., Matsufuji H. (2010). Stroke status evoked adhesion molecule genetic alterations in astrocytes isolated from stroke-prone spontaneously hypertensive rats and the apigenin inhibition of their expression. *Stroke Research and Treatment*.

[39] Klimaczewski C. V., Saraiva R. D. A., Roos D. H. (2014). Antioxidant activity of *Peumus boldus* extract and alkaloid boldine against damage induced by Fe(II)-citrate in rat liver mitochondria in vitro. *Industrial Crops and Products*.

[40] Abbas S. R., Sabir S. M., Ahmad S. D., Boligon A. A., Athayde M. L. (2014). Phenolic profile, antioxidant potential and DNA damage protecting activity of sugarcane (*Saccharum officinarum*). *Food Chemistry*.

[41] Kim H. K., Mendonça K. M., Howson P. A., Brotchie J. M., Andreazza A. C. (2015). The link between mitochondrial complex i and brain-derived neurotrophic factor in SH-SY5Y cells—the potential of JNX1001 as a therapeutic agent. *European Journal of Pharmacology*.

[42] Andreazza A. C., Wang J.-F., Salmasi F., Shao L., Young L. T. (2013). Specific subcellular changes in oxidative stress in prefrontal cortex from patients with bipolar disorder. *Journal of Neurochemistry*.

[43] Costa F., Dornelles E., Mânica-Cattani M. F. (2012). Influence of Val16Ala SOD2 polymorphism on the in-vitro effect of clomiphene citrate in oxidative metabolism. *Reproductive BioMedicine Online*.

[44] Mimaki M., Wang X., McKenzie M., Thorburn D. R., Ryan M. T. (2012). Understanding mitochondrial complex I assembly in health and disease. *Biochimica et Biophysica Acta—Bioenergetics*.

[45] Brazier-Hicks M., Evans K. M., Gershater M. C., Puschmann H., Steel P. G., Edwards R. (2009). The C-glycosylation of flavonoids in cereals. *The Journal of Biological Chemistry*.

[46] Law B. N. T., Ling A. P. K., Koh R. Y., Chye S. M., Wong Y. P. (2014). Neuroprotective effects of orientin on hydrogen peroxide induced apoptosis in SHSY5Y cells. *Molecular Medicine Reports*.

[47] Simirgiotis M. J., Schmeda-Hirschmann G., Bórquez J., Kennelly E. J. (2013). The Passiflora tripartita (banana passion) fruit: a source of bioactive flavonoid C-glycosides isolated by HSCCC and characterized by HPLC-DAD-ESI/MS/MS. *Molecules*.

[48] Yoo H., Ku S.-K., Lee T., Bae J.-S. (2014). Orientin inhibits HMGB1-induced inflammatory responses in HUVECs and in murine polymicrobial sepsis. *Inflammation*.

[49] An F., Yang G. D., Tian J. M., Wang S. H. (2012). Antioxidant effects of the orientin and vitexin in trollius chinensis bunge in D-galactose-aged mice. *Neural Regeneration Research*.

[50] Mathew S., Abraham T. E., Zakaria Z. A. (2015). Reactivity of phenolic compounds towards free radicals under in vitro conditions. *Journal of Food Science and Technology*.

[51] Popović M., Caballero-Bleda M., Benavente-García O., Castillo J. (2014). The flavonoid apigenin delays forgetting of passive avoidance conditioning in rats. *Journal of Psychopharmacology*.

[52] Hollman P. C. H., Katan M. B. (1999). Health effects and bioavailability of dietary flavonols. *Free Radical Research*.

[53] van Praag H., Lucero M. J., Yeo G. W. (2007). Plant-derived flavanol (−)epicatechin enhances angiogenesis and retention of spatial memory in mice. *The Journal of Neuroscience*.

[54] Rangel-Ordóñez L., Nöldner M., Schubert-Zsilavecz M., Wurglics M. (2010). Plasma levels and distribution of flavonoids in rat brain after single and repeated doses of standardized Ginkgo biloba extract EGb 761®. *Planta Medica*.

[55] Milbury P. E., Kalt W. (2010). Xenobiotic metabolism and berry flavonoid transport across the blood-brain barrier. *Journal of Agricultural and Food Chemistry*.

[56] Wong D. Y. S., Musgrave I. F., Harvey B. S., Smid S. D. (2013). Açaí (*Euterpe oleraceae* Mart.) berry extract exerts neuroprotective effects against *β*-amyloid exposure in vitro. *Neuroscience Letters*.

[57] Halliwell B. (2001). Role of free radicals in the neurodegenerative diseases: therapeutic implications for antioxidant treatment. *Drugs and Aging*.

[58] Scola G., Kim H. K., Young L. T., Salvador M., Andreazza A. C. (2014). Lithium reduces the effects of rotenone-induced complex I dysfunction on DNA methylation and hydroxymethylation in rat cortical primary neurons. *Psychopharmacology*.

[59] Guerra J. F. D. C., Magalhães C. L. D. B., Costa D. C., Silva M. E., Pedrosa M. L. (2011). Dietary açai modulates ROS production by neutrophils and gene expression of liver antioxidant enzymes in rats. *Journal of Clinical Biochemistry and Nutrition*.

[60] Masella R., Di Benedetto R., Varì R., Filesi C., Giovannini C. (2005). Novel mechanisms of natural antioxidant compounds in biological systems: involvement of glutathione and glutathione-related enzymes. *The Journal of Nutritional Biochemistry*.

[61] Lannuzel A., Höglinger G. U., Champy P., Michel P. P., Hirsch E. C., Ruberg M. (2006). Is atypical parkinsonism in the Caribbean caused by the consumption of Annonacae?. *Journal of Neural Transmission*.

